# Supplementary material for: Are carbon emissions trading and green financial instruments synergistic? -Comprehensive quantitative research based on content analysis
Source: PLoS One. 2024 Mar 7;19(3):e0298601. doi: 10.1371/journal.pone.0298601 (PMC10919601; doi:10.1371/journal.pone.0298601)
Supplement: S1 Data — (ZIP) [file pone.0298601.s001.zip › Data/地级市Z_F工作报告.zip!/║■─╧/║Γ╤⌠╩╨/└┤╫╘╣1⁄2╓┌║┼í╛┬φ┐╦╩2╛▌═°í┐_2015.docx]

各位代表：

现在，我代表市人民政府，向大会作政府工作报告，请予审查，并请各位政协委员和其他列席人员提出意见。

一、2014年工作回顾

过去一年，面对宏观经济下行、处理破坏选举案、调整产业结构等多重压力叠加的严峻形势，在市委的坚强领导下，在市人大、市政协的监督支持下，市政府团结带领全市广大干部群众，努力拼搏，攻坚克难，统筹推进稳增长、促改革、调结构、惠民生，保持了经济社会平稳健康发展。

(一)经济发展稳中有进。经济实力不断提升。全年实现地区生产总值2395.56亿元(初步核算，下同)，增长9.9%；完成财政总收入235.66亿元，增长11.2%；完成固定资产投资1767.01亿元，增长22.9%；实现社会消费品零售总额825.51亿元，增长12.8%，综合实力稳居全省第一方阵。产业结构不断优化。三次产业结构调整为15.2∶46.8∶38.0，第三产业比重提高1.5个百分点。粮食再获丰收，衡阳县、耒阳市被评为全国粮食生产先进县(市)，国家“蔬菜优质种苗繁育基地”落户我市，新增省级农业产业化龙头企业数量位居全省第一。工业经济企稳回升，规模工业增加值增长9.3%，高技术产业增加值占规模工业增加值的比重提高4.7个百分点，我市连续四届荣获“中国城市信息化50强”称号，常宁成为全省第二批特色县域经济(制造产业)重点县。三产增加值增长13.1%，对经济增长的贡献率达49%，创近年来最好成绩。接待国内外游客和实现旅游收入分别增长14.1%、20.2%，蔡伦竹海成功创建为国家4A级旅游景区，四明山获评国家森林公园。发展质量不断提高。单位GDP能耗、单位工业增加值能耗分别下降7.1%、11.5%；存贷比优化为51.3%，较年初提高7.85个百分点；城镇和农村居民人均可支配收入分别增长9.3%、11.5%；科技创新成效明显，衡变公司获国家科技进步一等奖，全市专利授权量达1900件。发展后劲不断增强。全市开工建设445个重点项目，完成投资823.13亿元，平均每天1.2个项目和2.26亿元投资落地，均创历史新高。南岳机场正式通航；吉衡铁路正式通车运营，怀邵衡铁路加快推进；湘江土谷塘航电枢纽(一期)开闸通航，湘江衡阳至株洲2000吨级航道工可获批，归阳千吨级码头启动征拆；娄衡高速、南岳高速东延线、武广衡山西站至南岳连接线加快建设；衡云、衡西干线等13条干线公路有序推进，交通融城步伐明显加快，“对外大联通、对内大循环”的立体交通基本成形。中国五矿水口山金铜、富士康(衡阳)工业新城、国家农业科技园、华耀城、崇盛国际中心、红星美凯龙购物广场等项目推进迅速，龙头项目的带动辐射能力不断增强。南华大学新校区、湖南高铁职院新校区(二期)、湖南工学院新校区(二期)和湖南环境生物职业技术学院扩建有序推进，市铁一中和市九中搬迁工程顺利开工，湖南交通工程学院成功升为本科院校；“三馆两中心”、市中心医院异地新建、常宁歌剧院等项目进展顺利，项目惠民力度不断加大。

(二)改革开放持续深化。重点改革稳步推进。商事登记制度改革扎实开展，全市新登记企业和注册资本分别增长70.8%、115.7%。企业改制攻坚克难，历时14年的国有工业企业改制基本完成，市本级集体企业改制接近尾声。财税体制改革初见成效，预算公开有序推进，市本级“三公”经费明显下降。农村综合改革成效明显，农村土地承包经营权确权登记颁证试点工作进展顺利，“一流转五服务”工作逐步推广，耒阳在全省率先实行农村土地承包经营权、农村房屋资产和农机具打包抵押贷款模式，常宁在全省率先成立林权交易管理中心，衡南入选“信息进村入户整建制推进试点县”，并乡(镇)工作在祁东、合村工作在衡阳县井头镇和长安乡完成试点。机制体制不断创新。市区两级管理职能调整持续深化，完成园林绿化体制改革。公交改革深入推进，公交公司股权及管理全面优化调整。市本级7大投融资公司现代企业制度基本建立。资本市场实现重大突破，天雁机械在主板成功上市，5家企业在“新三板”成功上市。对外开放不断扩大。衡阳综保区正式封关运行，衡山科学城开创高端创新型园区建设、招商新模式，铁路口岸建设扎实推进。着力把工业园区打造为对外开放的主平台，白沙洲工业园、松木经开区和祁东归阳工业园、耒阳循环经济产业园建设提速，西渡高新区核心片基础设施建设全面启动，省级及以上产业园区规模工业增加值占全市规模工业增加值的比重首次突破50%。招商引资来势喜人，14家世界500强企业来衡考察洽谈，承接重大产业转移项目18个，实际到位内资255.17亿元、增长12.5%，实际利用外资9.0亿美元、增长18%，实现外贸进出口总额29.43亿美元、增长62.4%。

(三)城乡面貌明显改观。围绕建设“宜居家园”目标，加快推进新型城镇化和新农村建设，城镇化率提高到48.5%。扩容提质成效明显。大力推进来雁新城、滨江新区建设，着力打造衡阳城市新名片；高标准实施衡州大道、船山大道等6条城市主干道两厢提质改造；衡桂高速常宁连接线、S315线衡阳县城段、107国道耒阳城区段、322国道衡南段完成提质升级；船山东路、二环路等30条城市主次干道加快建设；南湖公园(一期)竣工开园，东洲岛、酃湖公园、幸福河改造等全市人民翘首以盼多年的项目取得实质性进展，城市规模和品位不断提升。配套设施日趋完善。珠晖水厂厂区工程全面完成，衡阳县自来水公司6万吨新水厂加快建设；江东污水处理厂、城西污水处理厂(二期)、常宁松柏镇污水处理厂竣工，城区污水处理率达85%。管理水平不断提升。扎实开展“城市管理年”活动，创新渣土、园林绿化工程质量管理机制，城市重点部位摊点占道、户外广告杂乱、渣土污染等突出问题得到有效整治；拆违控违工作成效明显；稳步推进殡葬改革，中心城区乱搭灵棚治丧行为基本杜绝；积极开展中心城区烟花爆竹限放工作。人居环境不断改善。扎实推进湘江流域保护和治理“一号重点工程”，关停并转涉重金属污染企业111家，完成重金属污染源治理项目47个，一年完成养殖场关停搬迁省定的两年任务；完成人工造林34万亩，森林覆盖率达45.9%；全面开展农村环境卫生综合整治，集镇“五乱”现象得到有效遏制，美丽乡村建设有力推进。

(四)民生改善扎实有效。坚持财政收入改善民生的主导向，民生支出占财政总支出的比重达66.7%。16项30个为民办实事任务全部或超额完成。新增城镇就业7.6万人，农村劳动力转移就业6.2万人；企业退休人员养老金、被征地农民社保待遇分别实现“十连调”和“五连调”；开工建设保障性安居工程2.39万套，圆满完成省里下达的目标任务；托市粮收购量连续两年位居全省第一。一批联系和服务群众的“三最”问题得到有效解决，完成295个村农网改造，开工建设安置房118万平方米，中心城区商品房产权办理遗留问题逐步消化，解决50.3万农村人口的饮水安全问题，实现6万名低收入贫困人口脱贫。

(五)社会事业稳步发展。城区大班额问题逐步缓解，高考二本以上上线人数位居全省第二，未成年人社会主义核心价值观教育被评为全国先进。首次荣膺全国文化系统先进集体，衡山皮影戏成功申报为世界非物质文化遗产，常宁、耒阳分别被评为“中国民间文化艺术之乡”、“千年古县”；《衡阳市志(1978-2008)》正式出版。“单独两孩”政策有序实施，公共卫生服务体系不断完善，基本药物制度实施成果得到巩固。参赛省第十二届运动会取得历史最好成绩。海关、检验检疫服务外向型经济发展更加积极主动。

(六)社会大局和谐稳定。全年未发生重大以上安全生产事故，安全生产考核连续两年排名全省第一。严厉打击非法集资，守住了不发生区域性和系统性金融风险的底线，全市银行金融机构不良贷款比年初减少7.98亿元。创新信访“一岗双责”制，深入开展“大排查、大接访、大化解”活动；“平安衡阳”创建扎实开展，派出所建设治安防控工作成为全省样板；开通“12345”政府公共服务平台，受理并解决群众诉求15万余件。

(七)民主法制建设深入推进。自觉接受人大及其常委会法律监督、工作监督和政协民主监督，充分吸纳各民主党派、工商联和无党派人士的意见、建议，办理市人大代表议案1件、建议203件，市政协提案505件。依法治市扎实推进，“六五”普法深入开展，现行规范性文件得到全面清理。完成第九次村委会换届选举工作。

(八)政府自身建设不断加强。深入开展党的群众路线教育实践活动，扎实推进“发展环境优化年”和机关效能建设，干部作风持续好转，发展环境进一步改善。市县两级政府职能转变和机构改革工作加快实施，政务服务和政务公开深入推进；行政审批制度改革成效明显，市本级行政审批事项由373项清理至147项。行政监察、经济责任审计和社会监督不断加强。

国防动员和后备力量建设扎实开展。国防动员工作制度不断健全，民兵预备役调整初见成效。双拥共建和“双带双促”活动深入开展，军政军民团结局面进一步巩固。驻衡解放军、武警部队、民兵预备役部队为全市经济社会发展作出了新的贡献。

过去一年的成绩来之不易，值得倍加珍惜。这些成绩的取得，是市委正确领导的结果，是市人大、市政协监督支持的结果，是全市人民团结拼搏的结果。在此，我代表市人民政府，向全市人民；向人大代表、政协委员，离退休老同志；向各民主党派、工商联和无党派人士，各人民团体，驻衡部队、武警部队官兵和中央、省驻衡单位、高校、科研院所；向关心、支持衡阳建设发展的朋友们，表示衷心的感谢!

各位代表，在肯定成绩的同时，我们也要清醒地认识到，我市经济社会发展和政府工作还存在不少问题。一是受宏观经济形势严峻、市场需求疲软、结构调整、清理历史虚增等因素影响，部分经济指标增速没有达到年初预期。二是工业经济增速放缓，企业创新能力不强，战略性新兴产业支撑有限，税占比、存贷比仍然偏低，经济结构有待进一步优化。三是县域经济总体发展水平不高，七县市GDP、财政总收入平均增速比五城区分别低1.8个、4.2个百分点，小康指标的实现压力较大；城乡居民收入差距进一步拉大；各县市基础设施还比较薄弱，县级工业园区发展滞后。四是城市建设管理水平不高，控违拆违难、征地拆迁难、要素保障难等问题仍然困扰城市建设发展，城市基础设施的承载力与公共需求还不适应；城区交通拥堵还比较严重，背街小巷、城乡结合部环境治理仍然滞后。五是政府职能转变还不到位，简政放权还有较大空间，经济发展环境还不优，个别单位、少数工作人员“四风”问题还不同程度存在，为官不为、推诿扯皮、办事拖拉的情况时有发生。对此，我们将直面问题、不遮不掩，采取有力措施，逐步予以解决。

二、2015年工作安排

今年是全面深化改革的关键之年，也是全面推进依法治国的开局之年。做好今年的工作，必须紧密结合衡阳实际，准确把握和研判宏观经济形势。从国际经济大势看，世界经济仍处在国际金融危机后的深度调整期，但总体延续缓慢复苏态势，经济增速可能略有回升。从国内经济走势看，下行压力仍然较大，增速会持续放缓，但中央将保持宏观政策的连续性和稳定性，继续实施积极财政政策和稳健货币政策。从衡阳发展态势看，发展不足仍是我市最大的市情，但中高速增长将成为经济发展的常态，生产要素相对优势仍然存在，并将迎来深化改革、区域合作、新型城镇化等新机遇。全市上下必须把思想和行动统一到中央、省委和市委的决策部署上来，认清经济发展新常态的特征和要求，把握我市经济发展的现状与趋势，科学应对各类风险和挑战，牢牢把握经济发展主动权，努力夺取衡阳改革发展的新胜利。

2015年政府工作的总体要求是：全面贯彻党的十八大、十八届三中、四中全会和中央、省委、市委经济工作会议精神，应对大环境，营造小气候，适应新常态，谋求新发展。继续坚持稳增长、促改革、调结构、惠民生的工作方针，以改革为动力，以创新为支撑，以法治为保障，促进经济持续健康发展和社会和谐稳定。

综合考虑各方面因素，今年全市经济社会发展的主要预期目标是：地区生产总值增长9%以上，财政总收入增长11%以上，固定资产投资增长20%以上，社会消费品零售总额增长12.5%以上，进出口总额增长20%以上，规模工业增加值增长9.5%以上，城镇和农村居民人均可支配收入分别增长10%、11%以上，城镇登记失业率控制在4.5%以内，节能减排、安全生产等约束性指标全面完成省里下达的任务。

围绕上述总体要求和预期目标，我们将用心用力抓好以下八个方面的工作：

(一)更加突出稳增长和调结构的统筹协调

充分发挥内需的支撑作用。扎实推进450个以上、总投资4000亿元以上、年度计划投资900亿元以上的重点项目建设；充分发挥政府投资的引导作用，积极争取国省支持，加大市级投入。提升商贸、餐饮等传统消费，稳定发展房地产、汽车等大宗消费，培育发展信息网络、健康养老、文化旅游等新兴热点消费，打造多点支撑的消费增长格局。探索建立市级产业投资基金、中小微企业信贷、担保风险补偿基金，推广符合市情的PPP、资产证券化、股权融资、融资租赁等合作模式。全面提升煤、电、油、气、运等生产要素的保障能力。确保重大基础设施、产业转型升级、民生改善等项目用地需求。

加快工业经济提质增效升级。坚持新型工业化第一推动力，改造提升汽车零部件、盐卤化工、有色金属、输变电、钢铁冶炼等传统主导产业，着力培育电子信息、先进装备制造、新能源、新材料等战略性新兴产业，突出抓好中国五矿水口山金铜、富士康(衡阳)工业新城、共创光伏薄膜太阳能、深圳中航标准件、利德有陶瓷(三期)等项目建设，带动工业经济平稳增长。鼓励大众创业、万众创新，推进中小企业公共服务平台建设，加大对本土企业的支持和培育力度，引导中小微企业走“专精特新”发展之路。推动工业化和信息化融合发展，引进智能装备和成套设备，打造智能化工厂。推进国家信息消费城市试点，加快引进电商旗舰企业，大力发展电子商务，促进移动互联网、云计算、大数据与传统产业跨界融合。编制全市工业园区发展规划，实施创新创业园区“135”工程，全市11个省级及以上产业园区两年内新建300万平方米以上标准厂房，引进500家以上创新创业企业。稳妥推进白沙洲工业园、松木经开区扩园调规工作，大力推进白沙洲工业园创建国家新型工业化电子信息产业示范基地，积极开展水口山经开区国家级循环化改造试点，统筹抓好特变电工输变电产业园、汽车零部件产业园、界牌陶瓷工业园等专业园区建设，力争省级及以上产业园区规模工业增加值占全市规模工业增加值的比重达58%以上。

加快转变农业发展方式。稳定大宗农产品生产，确保粮食总产稳定在330万吨以上，健全粮食储备体系，保障粮食安全。实施高标准农田“三年行动计划”，扎实推进“五小”水利设施、病险水库除险加固、中小河流治理等重点项目建设。大力推进经营集约化、产销一体化、生产标准化、耕作机械化。调整优化农业产业结构，提质发展粮食、畜禽、油料、林木、果蔬、茶叶等支柱和特色产业，抓好烟叶生产，拓展休闲农业和生态循环农业，培育壮大10家农业产业化重点龙头企业，深入实施农产品加工业振兴规划，确保农产品加工业总产值增长21%以上。加快农业科技创新，扎实推进国家农业科技园创建工作。深入开展农产品质量安全专项整治行动。建立健全新型职业农民技能培训机制，积极培育新型农业经营主体。

大力发展现代服务业。抓好国家服务业综合改革试点验收工作，促进服务业转型升级。改造提升商贸流通、交通运输、家政服务、社区服务等生活性服务业，加快崇盛国际中心、美美世界等标杆项目建设，完善城区商业网点布局。积极发展研发设计、现代物流、会展等生产性服务业，大力推进华耀城、白沙物流园等项目建设，延伸现代高科技物流服务网络。优化金融生态环境，争创全省金融生态良好城市，大力发展村镇银行，规范小贷公司、融资性担保公司和民间融资中介机构，促成兴业银行、长沙银行等来衡设立分支机构，力争全市新增各类贷款200亿元以上。围绕打造大南岳旅游经济圈，提升景点、交通、酒店等设施建设投资力度和整体营销水平，加快天子山火文化主题公园、雨母山、东洲岛、莱茵水畔运动度假小镇等旅游项目建设，加速石鼓书院、常宁中国印山4A级旅游景区创建工作，力争全市接待国内外游客、实现旅游收入分别增长14%和16%。组织开展品牌商贸旅游交易活动，进一步繁荣市场、激活经济发展要素。

(二)以更大的勇气全面深化改革和扩大开放

深化重点领域改革。全面完成市县政府职能转变和机构改革，扎实推进事业单位登记制度改革和法人治理结构建设试点工作，积极探索市直部门履职履责评估办法。全面完成国有企业改制扫尾工作，不断加强国资监管；推进政企分开，基本完成党政机关、承担行政职能的事业单位与下属企业脱钩。深化财税体制改革，推行全口径预算，建立跨年度预算平衡机制，清理规范税收等优惠政策，加强专项资金管理，有效防范政府性债务风险；推进政府购买服务深入开展。大力推进工商登记制度改革和工商管理体制改革，抓好前置审批制度改革第二批试点工作。加快推进金融体制改革，完成城区联社改制组建衡阳农村商业银行；支持金融机构创新金融产品和服务方式；加快投融资公司集团化、专业化、实体化发展，加大政府债券和专项债券发行力度。深化农村综合改革，扎实推进“一流转五服务”工作，加快农村土地承包经营权、土地流转经营权确权登记颁证工作；扎实开展农村抵押贷款模式创新，组建农业信用担保公司，实现融资性担保体系县域全覆盖；开展供销合作社综合改革，构建农村新型流通经营网络，建设一批配送中心、乡镇集贸中心、直营店和城镇商业综合体；进一步理顺乡镇管理体制，有序推进并乡(镇)合村工作，完成中心城区社区整合。巩固提升殡葬改革成果，大力倡导文明节俭办丧。统筹抓好公车、司法、文化体制等改革工作，充分释放发展活力和改革红利。

强化创新驱动发展。整合财政贴息和专项引导资金，引导企业加大研发投入，年内新建省级以上工程技术研究中心、企业技术中心、重点实验室2家以上，新增高新技术企业10家以上。深化产学研合作机制，促成国家和省级研发单位、重点实验室来衡设立技术转移中心。实施专利和知识产权行动，加快“工业企业知识产权运用能力培育工程”建设，力争全市专利申请量增长15%以上。加快国家输变电装备制造高新技术产业化基地、国家高品质无缝钢管及深加工高新技术产业化基地建设，大力创建国家级创新型产业集群。推动移动互联网、电子信息、新材料、生物医药等领域的技术研发和运行模式创新，力争全年高新技术产业产值突破1100亿元。

扩大对外开放优势。抓好衡阳综保区项目入驻和配套，加快铁路口岸建设和航空口岸申报工作，积极发展临空临港经济。加快衡山科学城第一期样板区、投融资公共平台建设。加强出口示范基地和出口品牌建设，支持有实力的“雁字号”“走出去”，推进外贸破零倍增，发展壮大重点外贸企业、重点园区、重点县市，引导扶持中小企业开拓国际市场，不断增加开放型经济对经济增长的贡献份额。加强重大项目深度开发及策划包装，加大招商引资力度，通过构建产业基金、城市资源换投资、政府平台引导、小分队招商等办法，深化与央企及世界500强企业的对接合作，狠抓项目落地，确保全市实际利用外资突破10亿美元。

(三)全面提升城镇建设管理水平

加快基础设施建设。扎实推进怀邵衡铁路、瓦松铁路、娄衡高速、南岳高速东延线、湘江土谷塘航电枢纽、大浦通用机场、归阳和松柏千吨级码头、廖家湾变电站升压改造等项目，开工建设茶常高速祁东至常宁段、湘江衡阳至株洲2000吨级航道、大浦千吨级码头。大力推进船山东路、二环路、长丰大道北延、北三环等城市主干道建设，抓好解放大道、东风路等主干道两厢提质改造。加快推进来雁新城、滨江新区、酃湖公园、幸福河改造和衡南县城沿江风光带、衡阳县蒸西大道、祁东圣云大道、衡山开云生态新城、衡东武家山公园等项目建设，着力提升城市品质。完成演武坪水厂、城南水厂提质改造，完成铜桥港污水处理厂(二期)、角山污水处理厂(一期)和7个重点镇污水处理厂建设，开工建设松木和酃湖污水处理厂，力争2016年实现城市污水全收集、全处理，污泥无害化处理率达到100%，市本级污水再生利用率达到10%。

推进精细化管理。扎实开展全国文明城市创建活动。扩大政府购买服务的领域，将垃圾清运处置、设施管护、园林绿化、风光带养护等更多城管职能推向市场。加强空间景观管理，集中力量抓好临街建筑设施、户外广告、渣土扬尘污染、公园广场经营秩序等专项整治，有序推进中心城区烟花爆竹限放工作。大力推进立体绿化、城市照明远程智能监控系统、智能化机械式立体停车场建设。加强马路市场整治和农贸市场标准化改造。不断扩展和优化城乡公交线路，提升公交服务水平。加快“数字衡阳”建设，提升城市信息化管理水平。强力推进拆违控违工作，完善违法建设项目黑名单和曝光制度，严厉打击违法违规建设行为，严格规范农民建房建设管理。

不断提高城市经营效益。精心培育城市经营市场主体，调整城市基础设施公共产品的准入门槛，形成各类投资主体、企业、社会组织和市民共同参与的城市经营格局。加强土地整体规划利用管理，加大城市土地报批、收储力度，着力提高经营性用地单位面积收益。强化城市公共设施的运营和管理，积极将城市资本与资源推向市场，加大经营权、冠名权和特色文化等相关延利资本的商业化运作。

(四)着力激发县域经济发展活力

差异化发展主导产业。加快各县市产业兴城、产业融城步伐，严格按照全市产业规划布局，坚持“因地制宜、优势优先”的原则，积极引进战略投资者打造主导产业，做活、做通、做透产业链条，实现差异发展。加强县域工业园区建设，制订完善“飞地招商”的具体措施和税收分成办法，实现市直园区与各县市产业共建，各县市之间项目互补。

强化承载功能配套。不断加强县域公路、铁路、水路、航空等各种运输方式的有效衔接和融合，打开县城进出口门户，加快衡云、衡西干线和各县城通往集镇的主次干道建设。完成农村公路通畅工程550公里以上，基本实现全市所有具备条件的行政村通畅，加强农村公路提质改造和“安保工程”建设。积极参照市本级做法，完善城镇道路、公共交通、“两供两治”、配电网、地下综合管廊等市政设施建设，确保县以上城镇公共供水厂水质达标，县以上城镇污水处理率、生活垃圾无害化处理率分别达90%、95%以上。

加快小城镇建设。统筹城乡规划，精心编制乡镇总规和村庄布局规划，切实抓好乡镇政府所在地集镇的控规和专项规划编制，以规划引领中心镇、中心村建设。加大集镇土地开发和低效用地再开发力度，加快集镇发展和基础设施建设，对照2020年底全市集镇基础设施项目建设达到“10+2”的目标，每年重点抓好20个左右的小城镇示范点建设。推进农业转移人口市民化，保障农民在“家门口”充分就业创业。积极引导老乡回乡创业。保护特色古村落，强化产业引导和支撑，各县市每年重点选择12个乡镇连片推进新农村建设，打造一批美丽乡村。

(五)加快建设绿色宜居衡阳

强力推进环境综合治理。深入推进湘江流域保护和治理第一个“三年行动计划”，突出治理涉重金属、涉氨氮以及历史遗留污染。重点抓好松江、大浦、松柏等区域重金属污染治理与土壤修复，探索农作物种植结构调整。加强河道采砂、水上餐饮、运输船舶和港口码头污染防治，全面启动河道保洁工作。加快推进大气污染防治，扎实开展工业企业、机动车尾气和建筑工地扬尘污染防治，加大高污染燃料禁燃区10吨以下燃煤锅炉和黄标车、老旧车的淘汰力度，抓好PM2.5等六项空气质量数据发布及重污染天气监测预警应急工作。深入推进农村环境综合整治，继续抓好上型号水库严禁化肥养鱼工作，大力整治和取缔马路市场，全面启动农村生活垃圾处理五年计划，力争5年内90%的村庄垃圾得到处理，年内完成400个行政村的人居环境整治任务。

严格落实节能减排。鼓励企业引进战略投资者，在技术、工艺和管理上实现高位“嫁接”，加大落后产能的淘汰力度，狠抓钢铁、有色、化工、建材等重点行业的清洁生产及节能减排。大力推进合同能源管理，强化能源消费总量和强度双控制，探索建立水权交易制度，全面实施排污权有偿使用和交易制度。严格执行总量前置审批政策，全面落实环保“三同时”制度，着力加强水泥、造纸、钢铁、火电、集中式污水处理厂等重点行业污染治理设施的运行监管 侯卫东官场笔记。因地制宜发展绿色能源。

切实加强生态保护和建设。拓宽绿化空间，重点抓好高速公路、高速铁路、城市外环线、国省道沿线，湘江及其一级支流两侧和中、小型水库周边的造林绿化，鼓励县、乡公路两侧逐步实现绿化全覆盖，力争森林覆盖率达46.3%，森林蓄积量增长3.5%。全面加强森林资源保护，依法落实森林面积占补平衡制度，在重点区域实施禁伐限伐措施，严防严控森林火灾。着力实施矿山生态修复治理工程，深入开展矿山、尾矿库、渣场专项整治，推进裸露山体复绿。倡导生态文明理念，鼓励生态绿色出行。(六)尽最大努力保障和改善民生

坚持办好民生实事。以创业带动就业，深入实施“大学生创业引领计划”和高校毕业生千人创业工程，探索建立创业失败保障机制，抓好省级创业型城市验收工作；加快推进社会保障体系建设，全面启动事业单位养老保险改革，稳步推进医疗保险城乡统筹和居民医保门诊统筹，扎实抓好社会养老保险扩面征缴工作，完善被征地农民社会保障政策，加快建立统一的城乡居民养老保险制度；推进“两房两棚”建设，年内开工建设保障性安居工程4.3万套；解决39.34万农村人口的饮水安全问题；加强行政村配电网改造，年内完成415个村农网改造升级任务。

稳步提高居民收入。推进企业薪酬制度改革，深化机关事业单位工资和津贴补贴制度改革，研究实行县以下机关公务员职务与职级并行和乡镇事业单位人员工作补贴制度。依法保障劳动者基本权益，保护合法收入。加强和改进社会救助，进一步规范城乡低保工作，完善低保标准与物价上涨挂钩的联动机制。更多面向特定人口、具体人口实施精准扶贫，帮助5万名低收入贫困人口脱贫，统筹抓好移民后扶工作。

巩固安全生产工作成果。坚决贯彻落实省委、省政府遏制重特大事故八条断然措施，健全“党政同责、一岗双责、齐抓共管”安全生产责任体系，严格落实党政领导干部带队检查安全生产工作制度，打好小煤矿关闭、烟花爆竹整顿退出、危险化学品“进园入市”和油气管道安全四大攻坚战，实现全市安全生产形势持续稳定好转，确保不发生较大以上安全生产事故。

加强和改进社会治理。全面落实领导干部值班接访、定期约访下访和积案包案督办等制度，构建运行网上信访平台，积极引导人民群众依法理性表达诉求。健全重大决策社会稳定风险评估机制，注重舆情研判与舆论引导，着力防止和化解改革发展中出现的矛盾和风险。完善应急管理机制体制，提升突发事件应对和处置工作水平。进一步推进社区矫正规范化建设。加快建立覆盖全过程的食品药品监管制度，深入开展食品药品安全重点专项整治，以最严格的监管、最严厉的处罚、最严肃的问责，保证食品药品安全。加强社会治安综合防控体系建设，深化社区警务战略，强化专案侦破，打击毒品犯罪，保障人民群众生命财产安全。

(七)扎实办好社会事业

加快建设教育强市。实施中小学校布局规划“七年行动计划”和城区中小学化解大班额“三年行动计划”，加强义务教育合格学校建设，优化城乡义务教育资源均衡配置，着力解决进城务工人员随迁子女就学问题。努力提升高中教育质量，合理推进普职分流，促进高等教育发展，支持在衡高校项目建设。出台全市职业教育发展规划，加快实施中等职业教育攻坚计划。支持和规范发展民办教育。构建普惠性学前教育体系。深化教育人事制度改革，抓好质量评价和招生考试改革。完善校车安全管理制度，全面提升学校安全管理水平。

推动文化大发展大繁荣。扎实开展“雅韵雁城”“周末百姓剧场”“全民读书月”等文化惠民活动，推进市影剧艺术中心、图书馆新馆等公共文化设施建设。实施广播电视“户户通”“村村响”工程，统筹城乡基本公共文化服务标准化、均等化发展。鼓励扶持文艺生产，推出一批群众喜闻乐见的文艺精品。积极申报国家历史文化名城，启动衡阳抗战纪念城项目建设。促进文化与科技、旅游等多种业态融合发展。大力发展新闻出版、广播影视、文学艺术和市志、档案事业。

积极推进医药卫生事业。全面启动县市公立医院综合改革。进一步完善新农合制度，启动大病保险工作，巩固基本药物制度实施成果，推进城乡公共卫生服务均等化。继续落实城市医院对口支援基层医疗卫生机构制度。扎实抓好疫情监测和网络直报工作，加强重点传染病防控和突发公共卫生事件处置。重视贫困重性精神疾病患者救治救助。支持市中心医院、妇幼保健院异地新建和第三人民医院北院搬迁。协调推进中医药事业持续健康发展。努力构建和谐医患关系，严厉打击针对医院和医务人员的暴力违法犯罪行为。

统筹发展其他社会事业。坚持计划生育基本国策，促进人口长期均衡发展。广泛深入开展全民健身活动，启动承办省第十三届运动会、省第十届残运会的筹备工作，精心组织市第十届运动会，年内建成市体育中心“两馆”并投入使用。加快现代人民防空体系建设，推进人防融合发展。实施妇女儿童发展规划纲要，保障妇女儿童合法权益。添置养老服务床位，不断完善社会养老服务体系。关心和支持残疾人事业，帮助07岁残疾儿童实施抢救性康复。加快发展公益慈善事业。

(八)加快建设为民服务的法治政府

加强民主法制建设。自觉接受人大及其常委会监督，执行人大及其常委会的决议决定，自觉接受人民政协的民主监督，支持人民政协参政议政，认真办理人大代表建议和政协提案，积极采纳民主党派、工商联和无党派人士的调研成果。发展基层民主，全面开展社区“两化两体系”建设，做好第九次社区居委会换届选举工作。加强公共法律服务体系建设。支持司法机关依法独立行使职权。深入开展全民普法，提升社会法治化水平。进一步加强民族宗教工作。

加大简政放权力度。全面公开政府权力清单、责任清单和负面清单，继续做好行政审批事项的承接落实和取消下放，全面取消非行政审批事项，通过深化行政审批制度改革，优化行政审批流程，使我市成为行政审批项目少、行政办事效率高、行政运行成本低、行政过程透明的城市。

提升行政执法水平。深化行政执法体制改革，推广相对集中执法权，推进跨部门综合执法。严格落实行政执法人员持证上岗、资格管理和重大执法决定法制审核备案制度，深入推进“两法衔接”工作，规范公正文明执法，严格执行罚缴分离和收支两条线管理制度，最大限度减少行政自由裁量权。开展依法行政示范单位创建活动。

规范政府权力内部运行和监督。完善行政决策机制，坚持重大行政行为必经内部合法性审查，建立健全重大决策终身责任追究及责任倒查机制。推行政府法律顾问制度。全面推进政务公开，加快市政务服务中心整体搬迁工作。加强监督体系建设，金庸小说 支持和保障监察、财政、审计、法制等部门依法依规开展监督。完善行政复议制度和纠错问责机制，提高政府公信力和执行力。

持续加强和改进作风建设。巩固党的群众路线教育实践活动成果，自觉践行“三严三实”，加快形成作风建设长效机制。进一步优化发展环境，着力治理和解决办事难、乱检查、乱收费、乱处罚等群众反映强烈的问题。严肃廉政纪律，严格落实党风廉政建设责任制，坚决杜绝违法干预和插手工程、项目等行为，依法严厉查处腐败案件。

加强国防和后备力量建设。统筹推进军民融合深度发展，扎实抓好国防动员、民兵预备役、武警、应急动员、人防及军事设施保护工作。深化兵役制度改革，提高兵员质量。广泛开展全民国防教育和“双拥”共建活动，认真落实军转安置任务。

今年是全面完成“十二五”规划的收官之年，科学编制“十三五”规划是今年的一项重大任务。要从市情实际出发，深入研究经济新常态的新规律，准确把握面临的新机遇和新挑战，科学提出今后五年的发展目标和任务，使规划成为凝聚力量、指引发展的任务书、路线图。

各位代表，党的十八届四中全会吹响了全面推进依法治国的号角，做好今年的工作责任重大、意义重大。让我们在中共衡阳市委的坚强领导下，团结带领全市人民，解放思想，开拓进取，真抓实干，为加快全面建成小康衡阳而努力奋斗!
